# Phytochemical profiling and multi-target antibacterial in silico potential of Algerian wild sea buckthorn leaves

**DOI:** 10.1038/s41598-026-57022-2

**Published:** 2026-06-21

**Authors:** Fatima Zohra Chenni, Farouk Boudou, Fatima Zohra Ghanemi, Badra Bensabeur, Samia Chabane Chaouch, Samira Meziani, Jordi Saldo

**Affiliations:** 1 Biotoxicology Laboratory, Department of Biology, Faculty of Natural and Life Sciences, Djillali Liabes University, 22000 Sidi Bel Abbes, Algeria; 2 Department of Applied Molecular Genetics, Faculty of Natural and Life Sciences, University of Sciences and Technology of Oran – Mohamed-Boudiaf (USTO-MB), 31000 Oran, Algeria; 3 Natural Products Laboratory (LAPRONA), Department of Biology, Faculty of Natural Sciences, Life, Earth and Universe Sciences, Abou Bekr Belkaid, Tlemcen, Algeria; 4 Departament de Ciència Animal i dels Aliments, Facultat de Veterinària, Centre d’Innovació, Recerca i Transferència en Tecnologia dels Aliments (CIRTTA), XaRTA, TECNIO, MALTA-Consolider, Universitat Autónoma de Barcelona (Cerdanyola del Vallès), Barcelona, Spain; 5 Centro de Investigación de Alimentos (CIAL), Universidad UTE, Quito, Ecuador

**Keywords:** Sea buckthorn, Phytochemical profiling, Multi-target, PBP3, Molecular docking, Density functional theory (DFT), Molecular dynamics, Biochemistry, Chemical biology, Chemistry, Computational biology and bioinformatics, Drug discovery, Plant sciences

## Abstract

This study investigates the phytochemical profile and mechanistic bioactivity of sea buckthorn (*Hippophae rhamnoides* L.) leaves harvested from wild Algerian species. The research integrates quantitative HPLC profiling with in vitro and in silico assays to elucidate the molecular basis of its biological potential, specifically targeting the transpeptidase domain of Penicillin-Binding Protein 3. Two extracts (pure ethanol and 50:50 hydroethanol) were evaluated for their total phenolic, flavonoid, and tannin contents, along with antioxidant and antimicrobial activities. Key bioactive compounds identified through HPLC were subjected to Molecular Docking, Density Functional Theory, and 100 ns Molecular Dynamics (MD) simulations to assess their interactions with Penicillin-Binding Protein 3, as the primary target for molecular dynamics, while FtsZ and DNA Gyrase B were evaluated to assess multi-targeting potential. The hydroethanolic extract exhibited a higher yield (10.9%) compared to the ethanolic extract (3.7%), as well as superior levels of total phenolics (207.07 ± 0.94 mg GAE/g DW), flavonoids (4.19 ± 0.21 mg CE/g DW), and tannins (191.76 ± 1.55 mg CE/g DW). While the ethanolic extract showed higher antioxidant activity (IC_50_ of 0.147 mg/mL), the hydroethanolic extract displayed greater antimicrobial activity, particularly against *Staphylococcus aureus* (14.37 mm inhibition zone). HPLC analysis identified catechin (72.35 µg/g DW) and myricetin (6.34 µg/g DW) as the predominant compounds. In silico analysis revealed that while myricetin exhibited the highest initial binding affinity (− 8.1 kcal/mol) and the highest chemical reactivity, kaempferol demonstrated superior structural stability and compactness during the 100 ns MD simulations. The 7ONN-Kaempferol complex maintained high stability with a low root-mean-square deviation (RMSD, ~ 1.5–2.0 Å) and minimal residue fluctuations (RMSF < 0.8 Å). These findings provide a computational basis for the mechanistic link between specific flavonoid aglycones and the simultaneous disruption of bacterial cell wall synthesis, bacterial division, and DNA replication. This study highlights the potential of sea buckthorn leaves as a sustainable source of natural bioactive compounds with promising therapeutic applications.

**Supplementary Information:**

The online version contains supplementary material available at 10.1038/s41598-026-57022-2.

## Introduction

Natural products have been invaluable sources of molecules, widely used for centuries to treat various diseases. Their applications have extended into the cosmetic, food, and various other industries^1,2^. Plants, in particular, are valuable natural resources containing a large number of chemical compounds, including essential nutrients (vitamins, minerals, and fibers), and non-nutrient bioactive compounds such as phenolics, which exhibit diverse properties^3^ and applications^4^. Today, these compounds are prioritized due to their safety and potent antioxidant, antimicrobial and antifungal activities^5^. The exploration of such alternatives is currently a global priority due to the expansion of antimicrobial resistance (AMR) and toxicity concerns regarding synthetic antioxidants like BHA and BHT^6^. *Sea* buckthorn (*Hippophae rhamnoides L*.), an important member of the genus *Hippophae*, belonging to the *Elaeagnaceae* family, has recently gained significant worldwide attention as a valuable natural resource^7–9^. The fruits, leaves, and roots of *sea buckthorn* are rich sources of phytochemicals, including diverse secondary metabolites, such as phenolic compounds (e.g., flavonoids like quercetin, kaempferol, isorhamnetin, and catechins, as well as phenolic acids), organic acids, phytosterols, triterpenoids, coumarins, proanthocyanidins, and volatile compounds^10^. The highest phenolic content has been reported in the leaves, followed by the fruit, pulp, and berries^11^. While the berries are commonly used in the food industry and traditional medicine, sea buckthorn leaves remain an underutilized byproduct, despite containing the highest phenolic concentrations compared to the pulp or berries. However, existing studies on leaves remain descriptive, lacking deep mechanistic explanation of how their specific bioactive compounds interact with biological targets. Phenolic compounds, especially flavonoids and tannins, are increasingly used as natural agents in the food, cosmetic, chemical, and pharmaceutical industries^12^. These natural compounds have been reported to possess various biological properties, including antioxidant, antibacterial, and anti-inflammatory activities, which display diverse health-promoting effects. While the general profile and bioactivity of *Hippophae rhamnoides* L. are documented. This research provides the first integrated in vitro and in silico assessment of a specific wild population from Algeria. Recently, the bioactive potential of Algerian sea buckthorn has gained renewed interest^13^. Nevertheless, a critical need remains for molecular level validation to bridge the gap between phytochemical profiling and clinical application. To combat antimicrobial resistance, identifying natural inhibitors of Penicillin Binding Protein 3 (PBP3), a vital enzyme for bacterial septation, is of paramount importance. In this context, we hypothesize that the unique phenolic profile of Algerian sea buckthorn contains specific compounds that act as potent inhibitors of this target. Unlike traditional descriptive screenings, this study employs an integrated multi-scale approach : HPLC profiling to identify bioactive markers, Density Functional Theory (DFT) to evaluate chemical reactivity, and 100 ns Molecular Dynamics (MD) simulations to assess the structural stability and conformational flexibility of ligand–protein complexes under physiological conditions to provide molecular-level validation of these interactions, bridging the gap between raw phytochemical data and biological mechanism. Therefore, the present work aims to: (i) investigate the chemical composition of ethanolic and hydroethanolic sea buckthorn leaf extracts; (ii) compare their antioxidant and antimicrobial properties; (iii) identify the phytochemical composition of the most active extract using HPLC analysis; and (iv) conduct a comprehensive molecular modeling study (docking, DFT, and MD) to explore its therapeutic potential. The integrated strategy is increasingly employed to validate the mechanisms of action and drug-likeness of bioactive natural compounds. In this context, modern drug discovery emphasizes the synergy between PASS prediction and ADMET profiling to efficiently screen natural libraries. Such a multi-faceted approach is essential to validate the pharmacotherapeutic potential and safety profiles of these bioactive natural compounds^14,15^.

## Materials and methods

### Chemicals and reagents

All solvents, reagents, and standards used in the current study were of analytical grade. 1,1- diphenyl-2-picryl-hydrazyl (DPPH), and Folin-Ciocalteu reagent were purchased from Sigma-Aldrich. Gallic acid, catechin, and vanillin were obtained from.

Biochimica. Chemicals used for HPLC analysis were purchased from Sigma-Aldrich. The following standards were used for HPLC: epicatechin, quinic acid, vanillic acid, cinnamic acid, coumarin, quercetin, ascorbic acid, linoleic acid, sinapic acid, gallic acid, butyric acid, caffeic acid, and nicotinamide.

### Plant material and extract preparation

The leaves investigated in this study were collected in autumn 2023 from a wild population located near the coast of Tipaza (Sidi Rached), in the central north of Algeria, at the geographical coordinates 36°33′45″ N and 2°32′00″ E. Permissions to collect sea buckthorn samples were obtained from the institutional authorities and the collection was conducted in accordance with national guidelines. The leaves were removed from the twigs and berries and cleaned with distilled water. The formal identification of the sea buckthorn (*Hippophae rhamnoides* L.) plant material was undertaken by specialists from the Faculty of Natural and Life Sciences at Djilali Liabes University. A voucher specimen with the collection number FC-01 has been prepared and stored in the research collection of Djilali Liabes University. The collected material has been deposited in the herbarium at the laboratory of the Institute of Agronomy. For the preparation of the two extracts, 20 g of dried samples were mixed with 100 mL of 96% ethanol (ethanolic extract) and with 100 mL of a 50:50 mixture of 96% ethanol and distilled water (hydroethanolic extract). The mixture was filtered using Whatman No.1 filter paper and concentrated under reduced pressure at 40 °C using a rotary evaporator. The dry residue was then dissolved in ethanol for further analysis.

### HPLC phytochemical profiling

Analysis of phenolic compounds was carried out using a High-Performance Liquid Chromatography (HPLC) system (Young Lin Instrument Co., Ltd. YL-clarity 9100) equipped with a UV–Vis detector and a universal injector. The separation was performed on a 250 × 4.6 mm, C18 column with a particle size of 5 µm maintained at 25 °C. The mobile phase consisted of Solvent A (water with 1% formic acid) and Solvent B (acetonitrile). The flow rate was kept at 1 mL/min. The injection volume of the extract was 20 µL, and peaks were detected in scan mode at a UV detection wavelength of 254 nm. Phenolic compound separation was achieved using a 37 min linear solvent gradient, as follows (percentage of Solvent A): 0 min 90% A, 5 min 80% A, 25 min 20% A, 30 min 20% A, 32 min 90% A, 37 min 90% A. Peaks were identified by comparing their relative retention times and UV spectra with those of 14 reference standards. Linearity was established by calculating the coefficient of determination (R^2^) from calibration curves of each standard. The limit of detection (LOD) and limit of quantification (LOQ) were determined by the signal-to-noise ratio (S/N) method, established at S/N = 3 and S/N = 10, respectively.

### Total phenolic content (TPC)

The TPC of the prepared ethanolic and hydroethanolic extracts was quantified by a spectrophotometric method at 765 nm according to^16^ with some modifications using the Folin-Ciocalteu assay. Extraction of phenolics was performed using 70% ethanol at room temperature as previously described. TPC was calculated from a standard curve of gallic acid (0–3 mg/mL). The linear regression equation for the calibration curve was y = 0.0027x + 0.008, with a coefficient of determination (R^2^) of 0.9927.

Results are expressed as mg of gallic acid equivalents per gram of dry weight (mg GAE/g DW).

### Total flavonoids content (TFC)

TFC was determined by the aluminum chloride colorimetric method as described by^17^. Briefly, 500 µl of the hydroethanolic extract was mixed with 150 µL of 5% sodium nitrite solution and 2 mL of distilled water. After 6 min, 150 µL of 10% aluminum chloride solution and 2 mL of 1 M sodium hydroxide solution were added. The final volume was adjusted to 5 mL with distilled water. Absorbance was measured at 510 nm. The calibration curve was prepared in the same manner using 0.04 to 0.36 mg/mL catechin. The linear regression equation for the calibration curve was y = 0.1772x + 0.0092, with a coefficient of determination (R^2^) of 0.9988. Results are expressed as mg of catechin equivalents per gram of dry weight (mg CE/g DW).

### Total tannins content (TTC)

The total content of tannins expressed as mg of catechin equivalents (CE) per gram of dry weight of sea buckthorn leaves, was determined by a colorimetric method using the vanillin/HCl method of^18^ with some modifications. A volume of 0.4 mL of extract was added to 3 mL of vanillin (1%) and 1.5 mL of 37% HCl. After 15 min of incubation at 30 °C, the absorbance was measured at 500 nm. The total tannin content was quantified using the same catechin standard curve applied for TFC (y = 0.1772x + 0.0092, R^2^ = 0.9988).

### DPPH radical scavenging activity

The antioxidant potential of the sea buckthorn extracts was assessed using 1,1- diphenyl-2-picryl-hydrazyl (DPPH) according to the method of^19^. Briefly, the DPPH solution was prepared by dissolving 4 mg of DPPH powder in 100 mL of methanol. Then, 1950 µL of DPPH was added to 50 µL of various concentrations of the extracts. The absorbance of samples was measured at 515 nm. Ascorbic acid and gallic acid were used as positive controls. The ability to scavenge DPPH radicals was calculated as follows:

$$\% \;DPPH\;radical\;scavenging\;activity = [A\__{control} - A\__{sample} /A\__{control} ]*100$$

The IC_50_ (concentration providing 50% inhibition) of extracts and standards was determined using regression curves in the linear range of concentrations.

### Ferric reducing antioxidant power

The reducing power (FRAP assay) of the extracts was determined as described by^20^ with some modifications. Briefly, 1 mL of the extract was mixed with 2.5 mL of 0.2 M phosphate buffer solution (pH 6.6) and 2.5 mL of 1% potassium ferrocyanide solution. The mixture was incubated at 50 °C for 20 min. After cooling down to room temperature, 2.5 mL of TCA (trichloroacetic acid) (10%) was added to stop the reaction. After centrifugation for 10 min at 3000 rpm, 2.5 mL of the supernatant was added to 2.5 mL of distilled water and 0.5 mL of ferric chloride. The absorbance was measured at 700 nm using water as a blank. Ascorbic acid was used as standard, and reducing power was expressed as EC_50_, which is defined as the effective concentration at which the absorbance is equal to 0.5.

### Antimicrobial activity

For the evaluation of antimicrobial activity, ethanolic and hydroethanolic extracts were tested against *S. aureus* and *E. coli*. Bacterial cultures were activated in nutrient broth at 30 °C for 18 h, and adjusted to approximately 1.5 × 10^8^ CFU/mL^21^. Each bacterial inoculum was swab-streaked on Mueller–Hinton agar as described by^22^. Wells of 6 mm in diameter were cut into the agar, and 30 μL of the two extracts were tested. Incubation was performed at 37 °C for 24 h. Antibacterial activity was assessed by measuring the diameter of the inhibition zone. Gentamicin (50 µg) and Ceftazidime (30 µg) were used as positive controls to validate the assay, while 96% ethanol was used as a negative control. Although agar diffusion is a semi-quantitative method, it remains a widely accepted and standardized assay for antimicrobial screening, providing reliable comparative information on antibacterial activity under controlled conditions.

#### Statistical analysis

All analyses were performed in triplicate, and the results were presented as means ± standard deviation (SD). The differences between the two extracts were analyzed using one-way or two-way analysis of variance (ANOVA) followed by Tukey’s post-hoc test to determine significant differences between the extracts using IBM SPSS Statistics version 24 . Additionally, the correlation between phytochemical content and biological activities was evaluated using Pearson’s correlation coefficient (r) calculated with GraphPad Prism version 8.0.2. *p* value ˂0.05 was considered statistically significant.

#### Target protein selection and molecular docking

The molecular docking study aimed to evaluate the inhibitory potential of selected sea buckthorn phytochemicals against microbial target proteins relevant to the pathogenicity of the tested strains. The Penicillin-Binding Protein 3 (PBP3) transpeptidase domain from *E. coli* (PDB ID: 7ONN) was selected as the representative molecular model for this study. PBP3 is an essential transpeptidase directly involved in peptidoglycan cross-linking and cell division. Since the catalytic transpeptidase domain is functionally conserved across different bacterial species, 7ONN serves as a relevant molecular target to explore the fundamental molecular interactions and inhibitory potential of sea buckthorn phytochemicals against the peptidoglycan biosynthetic pathway in both Gram-negative and Gram-positive bacteria^23^. Furthermore, PBP3 is a high priority drug target due to its accessibility in the periplasm and the absence of homologous proteins in humans, which minimizes the risk of potential side effects. To further evaluate the multi-targeting potential of sea buckthorn phenolics, two additional bacterial proteins were selected. The first one is a cell division protein FtsZ (PDB ID: 1W5A), and DNA Gyrase B (PDB ID: 1KZN), vital for DNA replication and structural maintenance. Targeting these diverse pathways alongside PBP3 provides a comprehensive understanding of the synergistic antibacterial mechanism of the identified compounds. The ligands, identified via HPLC analysis of sea buckthorn extracts, included ascorbic acid (PubChem CID: 54,670,067), caffeic acid (PubChem CID: 689,043), catechin (PubChem CID: 9064), cianidanol (PubChem CID: 9064), coumarin (CID_323), epicatechin (CID_72276), gallic acid (CID_370), kaempferol (CID_5280863), and myricetin (CID_5281672), with their 3D structures retrieved from PubChem.

Molecular docking simulations were performed using AutoDock Vina (v1.5.7). The intermolecular interactions were analyzed and visualized using *BIOVIA Discovery Studio Visualizer, PyMOL, and LigPlot*** + **, allowing the identification of key binding features, including hydrogen bonds, hydrophobic contacts, and other stabilizing interactions within the active site. The receptors were prepared by removing water molecules and co-crystallized ligands, followed by the addition of polar hydrogens, and assignment of Kollman charges, and then conversion to the PDBQT format. Ligand preparation involved geometry optimization at physiological pH (7.4), addition of Gasteiger charges, assignment of rotatable bonds, and conversion to PDBQT format. To ensure the reliability of the multi-target docking study, the docking protocol was validated for each target protein. For the primary model (7ONN), the co-crystallized ligand (VL5) was re-docked into the active site, yielding a best-pose RMSD of 0.355 Å. Similarly for 1W5A and 1KZN , protocol validation was performed by re-docking their respective native inhibitors. In all cases, the calculated Root Mean Square Deviation (RMSD) was found to be ˂ 2.0 Å, confirming the accuracy of the search spaces and parameters.

The docking search space (grid box) for the 7ONN protein was centered at X = 42.569, Y = 35.819, Z = 37.227 Ǻ with a size of X = 68.639, Y = 52.625, Z = 56.267 Ǻ and an exhaustiveness of 8. To validate the docking protocol, the co-crystallized ligand was re-docked into their active sites. The calculated Root Mean Square Deviation (RMSD) was found to be ˂ 2.0 Å, confirming the accuracy of the search space and parameters. For the multi-target docking, the search spaces were specifically defined to encompass the active sites of each target. The box of FtSZ (1W5A)^24^ was centered on the interphase binding pocket, and for DNA Gyrase B (1KZN)^25^, the grid was focused on the ATP-binding site. All docking simulations were performed with an exhaustiveness of 32 to ensure high-precision binding energy estimations.

Molecular docking simulations predicted binding affinities and interaction patterns of the ligands with the proteins’ active sites after structural optimization. The interactions were analyzed and visualized using BIOVIA Discovery Studio Visualizer 2021 and LigPlot + v2.2, revealing key binding features such as hydrogen bonds and hydrophobic interactions.

The quantum chemical properties of the selected ligands were evaluated using.

Density Functional Theory (DFT) at the B3LYP/631(d,p) level of theory with the Gaussian 09W software package. The solvent environment was simulated using the Polarizable Continuum Model (PCM) for water. A frequency analysis was performed after geometry optimization to confirm the absence of any imaginary frequency, verifying the stability of the optimized geometry. GaussView was employed for molecular visualization and orbital analysis. The optimized molecular structures were used to calculate essential electronic properties, including the energies of the highest occupied molecular orbital (HOMO) and the lowest unoccupied molecular orbital (LUMO). From these, the energy gap (Eg), calculated as the difference between the HOMO and LUMO energies, providing insights into the ligands’ base stability. Additionally, global hardness, global softness, chemical potential, and electrophilicity index were calculated to characterize the chemical reactivity and stability of the ligands. Visualizations of the HOMO and LUMO orbital distributions were also generated to depict electron density locations within these frontier orbitals.

#### Molecular dynamics analysis

Based on the binding free energy values obtained from the multi-target docking study, ligands with the lowest binding free energy, indicative of stronger and more significant interactions with the target proteins, were selected for molecular dynamics (MD) simulations to validate the predicted interactions and assess dynamic stability. The integration of MD simulations is essential for validating the robustness and stability of ligand–protein complexes under simulated physiological conditions^26,27^. While binding free energy was primarily estimated using Vina scores, the dynamic stability and energetic consistency of the complexes were assessed through 100 ns trajectories, providing a time-dependent validation of binding stability**. **The 7ONN-ligand complexes were prioritized for 100 ns MD simulation to serve as a high-resolution model for validating complex stability and conformational flexibility under physiological conditions, providing a mechanistic insights for the overall multi-target inhibitory profile. The simulations were performed using the GROMACS 2023-GPU package with the CHARMM36m force field. Each selected complex was solvated in a triclinic box using TIP3P water models and neutralized by adding *Na*^+^ and *Cl*^*-*^ ions at a concentration of 150 mM. Energy minimization was performed to eliminate steric clashes and optimize the system geometry, followed by equilibration under NVT (constant volume and temperature) and NPT (constant pressure and temperature) ensembles for 2 ns each. The production phase was conducted for 100 ns at 300 K and 1 bar. The molecular dynamics trajectories were analyzed to evaluate global protein behavior, local backbone flexibility, and the stability of protein–ligand interactions over time. While docking explored the multi-target breadth across PBP3, FtsZ, and DNA Gyrase B, 100 ns MD simulations were focused on the PBP3-kaempferol complex to establish a high resolution of stability for the primary inhibitory pathway. Key parameters, such as root-mean-square deviation (RMSD), root-mean-square fluctuation (RMSF), and radius of gyration (*R*_g_), were monitored to confirm the robustness of the predicted binding modes and assess complex stability. The long-term stability observed in these trajectories serves as a reliable indicator of binding strength, overcoming the lack of explicit MM-PBSA/GBSA binding free energy calculations.

#### Docking protocol validation

The reliability of the docking protocol was validated through a redocking analysis of the co-crystallized ligand VL5 within the active site of 7ONN using AutoDock Vina. The search space was centered at coordinates (31.796, 12.781, − 0.499) Å with dimensions of 37.337 × 31.126 × 32.339 Å^3^, ensuring full coverage of the binding pocket. Docking was performed with an exhaustiveness of 32, generating 20 binding modes within an energy range of 4 kcal/mol.

**Fig. 1:**
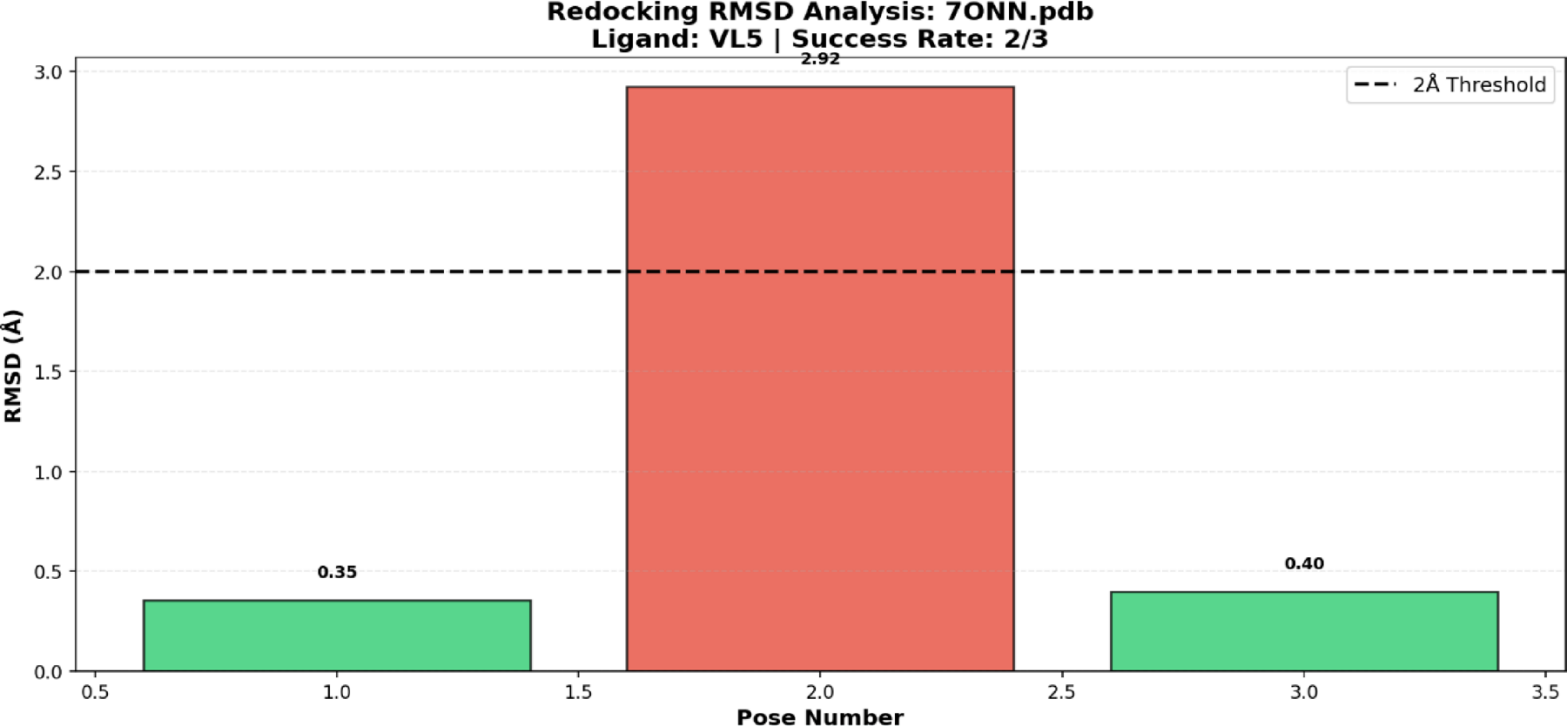
Redocking RMSD analysis of the crystallized ligand (VL5) within the 7ONN binding pocket.

The redocking results demonstrated a strong agreement between the predicted and crystallographic ligand poses. Among the generated conformations, two out of three poses (66.7%) exhibited RMSD values below the accepted validation threshold of 2.0 Å, confirming the robustness of the docking setup. The best-ranked pose (Pose 1) showed an RMSD of 0.355 Å, indicating near-perfect superposition with the native ligand orientation. Pose 3 also showed a highly accurate prediction with an RMSD of 0.398 Å. Only one pose exceeded the RMSD threshold (2.924 Å), suggesting minor alternative binding conformations (Fig. 1).

The mean RMSD (1.226 Å) and median RMSD (0.398 Å) further confirm the accuracy and reproducibility of the docking protocol. The very low best RMSD value (< 0.5 Å) demonstrates excellent predictive power, as values below 2.0 Å are generally considered successful for docking validation.

**Fig. 2:**
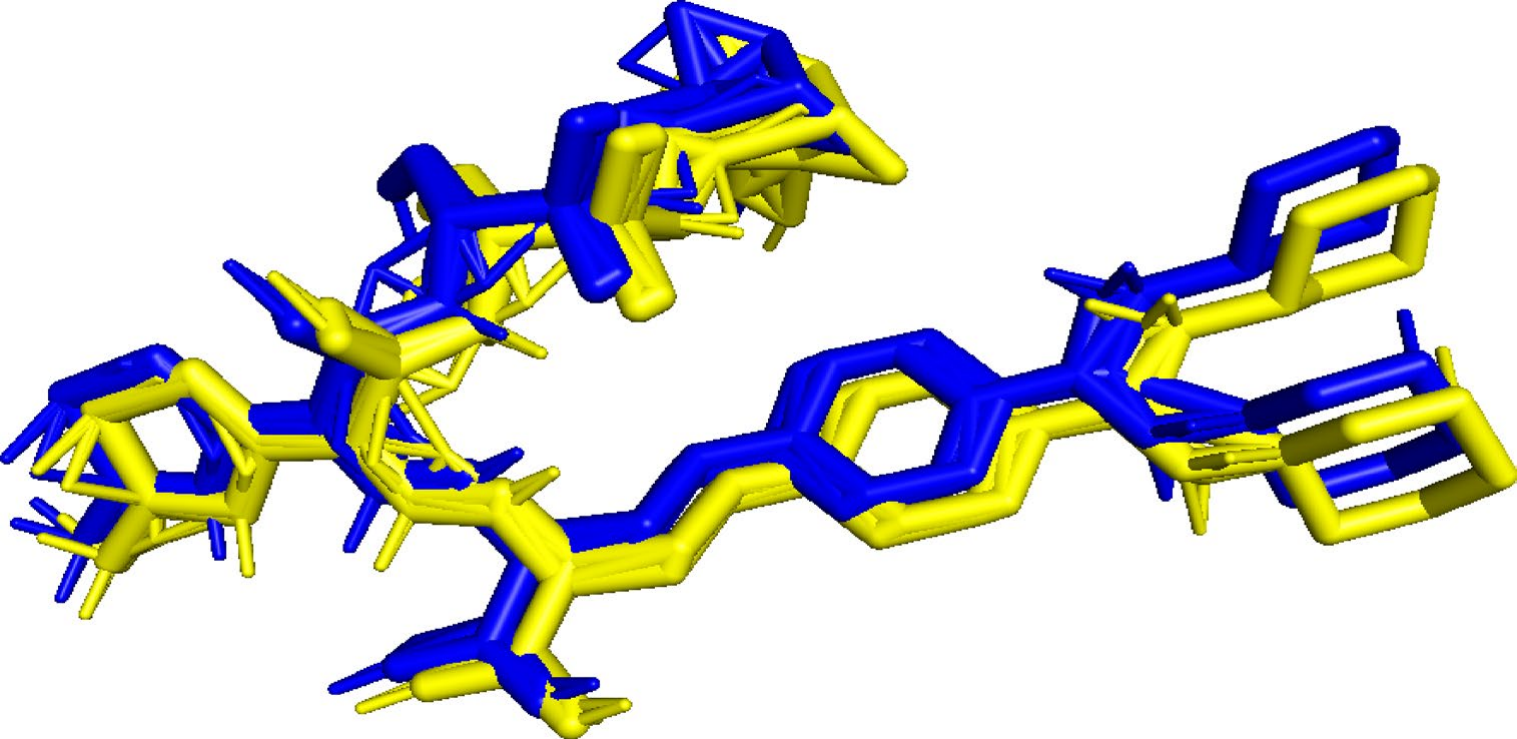
Structural superimposition of the native ligand VL5 and the best-docked pose.

Overall, the redocking analysis confirms that the selected grid parameters and docking settings are appropriate and reliable for subsequent docking studies of novel ligands against 7ONN (Fig. 2).

RMSD values of the top three docked poses are presented relative to the crystallographic conformation. The dashed horizontal line represents the validation threshold of 2 Å. Two poses (Pose 1 and Pose 3) fall below this threshold, confirming successful redocking (success rate: 2/3).

The co-crystallized native ligand VL5 (blue) is superimposed with the best-redocked pose (yellow) within the binding pocket of PBP3 (PDB ID:7ONN). The strong structural overlap, evidenced by a low RMSD value of 0.355 Å, validates the accuracy of the docking protocol.

### Ethics statement

All experimental research and field studies on plant material complied with relevant institutional, national, and international guidelines and legislation.

## Results and discussion

### Extraction yields

Results revealed an extraction yield of 10.9% in the hydroethanolic extract, while the ethanolic extract was found to be 3.7%. These results indicated that hydroethanolic extraction was more effective compared to ethanolic extraction. The extracts were obtained with 96% ethanol and a 50% ethanol/water mixture, respectively. Researchers have reported that solvent polarity has a considerable effect on extract yield^28^, and these results suggest that the addition of water to ethanol (hydroethanolic solution) allows much more extraction than using pure ethanol. Water increases the solvent’s polarity, which makes it more effective for extracting the bioactive compounds. These results are confirmed by other studies which reported that ethanol/water solvents were more efficient for extracting phenolic compounds than when water or ethanol were used alone. Similar results were also reported by other researchers, indicating that ethanol/water solvent is particularly suitable for obtaining extracts rich in polar phenolic components, especially flavonoids with high content. This may be because aqueous solvents are suitable for extracting much more bioactive compounds with high polarity; ethanol or ethanol/water solvent is suitable for extracting bioactive compounds with a wide polarity range. Thus, these results imply that the ethanol/water solvent may provide higher concentrations of total phenolics and flavonoids, and consequently may show better capacity as a free radical scavenger^29,30^.

### Total phenolic, flavonoid and tannins contents

As shown in Table 1, the quantitative analysis revealed a significant influence of solvent polarity on the recovery of bioactive molecules. The hydroethanolic extract showed significantly higher levels of TPC (207.07 ± 0.94 mg GAE/g DW) and TFC (4.19 ± 0.21 mg CE /g DW) compared to the ethanolic extract (*p* ˂ 0.05). Regarding total tannins, the hydroethanolic extract showed also a slightly higher content with a value of 191.76 ± 1.55 mg CE/g DW, compared to the ethanolic extract with a value of 188.24 ± 0.99 mg CE/g DW. The considerable amounts of bioactive compounds observed in this study are in accordance with previous reports^31,32^, who reported TPC values around 198.61 mg GAE/g. However, TFC content was found to be 30-fold higher compared to the TFC found in the hydroethanolic extract, and 50-fold higher in the ethanolic extract. In contrast, this result is not in line with that reported by^33^, who found lower TPC ranges (65 to 92 mg GAE/g) and 13 mg/g condensed tannins. Likewise,^7^ reported total polyphenolic content ranging from 41.60 to 48.12 mg GAE/g, while the content of flavonoids ranged from 31.53 to 36.58 mg QE/g. The study carried out by^31^, indicated higher total phenolic and flavonoid contents in the hydroethanolic leaf extract 56.28 mg GAE/g dry leaf and 20.76 mg RE /g dry leaf, respectively, compared to the values found in the aqueous leaf extracts 40.5 mg GAE/g dry leaf and 15 mg RE/ g dry leaf. In the study by^34^, the TPC of sea buckthorn leaves from Poland ranged from 7.06 to 10.69 mg/g DW.^32^ in their study found that leaves extracted by ethanol contain 165.76 mg/g total phenols, 47.76 mg/g total flavonoids, and 1.32 mg/g condensed tannins. From the above results and in agreement with literature, the levels of phenolic compounds, mainly flavonoids, phenolic acids, and tannins, vary significantly, depending on various factors such as extraction methods, solvent types, solvent/sample ratios, plant varieties, harvesting time, genotypes, and the specific anatomical parts used^16,32,35,36^. In our study, both extraction parameters and environmental conditions influence the phytochemical profile of Algerian sea buckthorn leaves. In this situation, the accumulation of bioactive compounds increases the phenolic levels in the leaves as a photoprotective mechanism^37^. Based on the superior total phenolic and flavonoid compounds yield and antimicrobial potency of the hydroethanolic extract, it was selected as the representative sample for the detailed HPLC profiling to identify the key bioactive markers responsible for these activities.

**Table 1:** Extraction yields and quantitative analysis of total phenolic, flavonoid and tannin contents of sea buckthorn leaf extracts.

Extract	Yield (%)	TPC (mg GAE/g DW)	TFC (mg CE/g DW)	Tannins (mg CE/g DW)
Ethanolic extract	3.7 ± 0.25^a^	183.31 ± 0.03^a^	2.54 ± 0.05^a^	188.24 ± 0.99^a^
Hydroethanolic extract	10.9 ± 0.58^b^	207.07 ± 0.94^b^	4.19 ± 0.21^b^	191.76 ± 1.55^a^

Values are expressed as mean ± SD (n = 3).

Means within the same column, followed by different superscripts letters indicate statistically significant differences $p\le 0.05$ as determined by one-way ANOVA followed by Tukey’s post-hoc test.

TPC, Total Phenolic Content; GAE, Gallic Acid Equivalent; TFC, Total Flavonoids Content; CE, Catechin Equivalents.

### Antioxidant activity

**Fig. 3:**
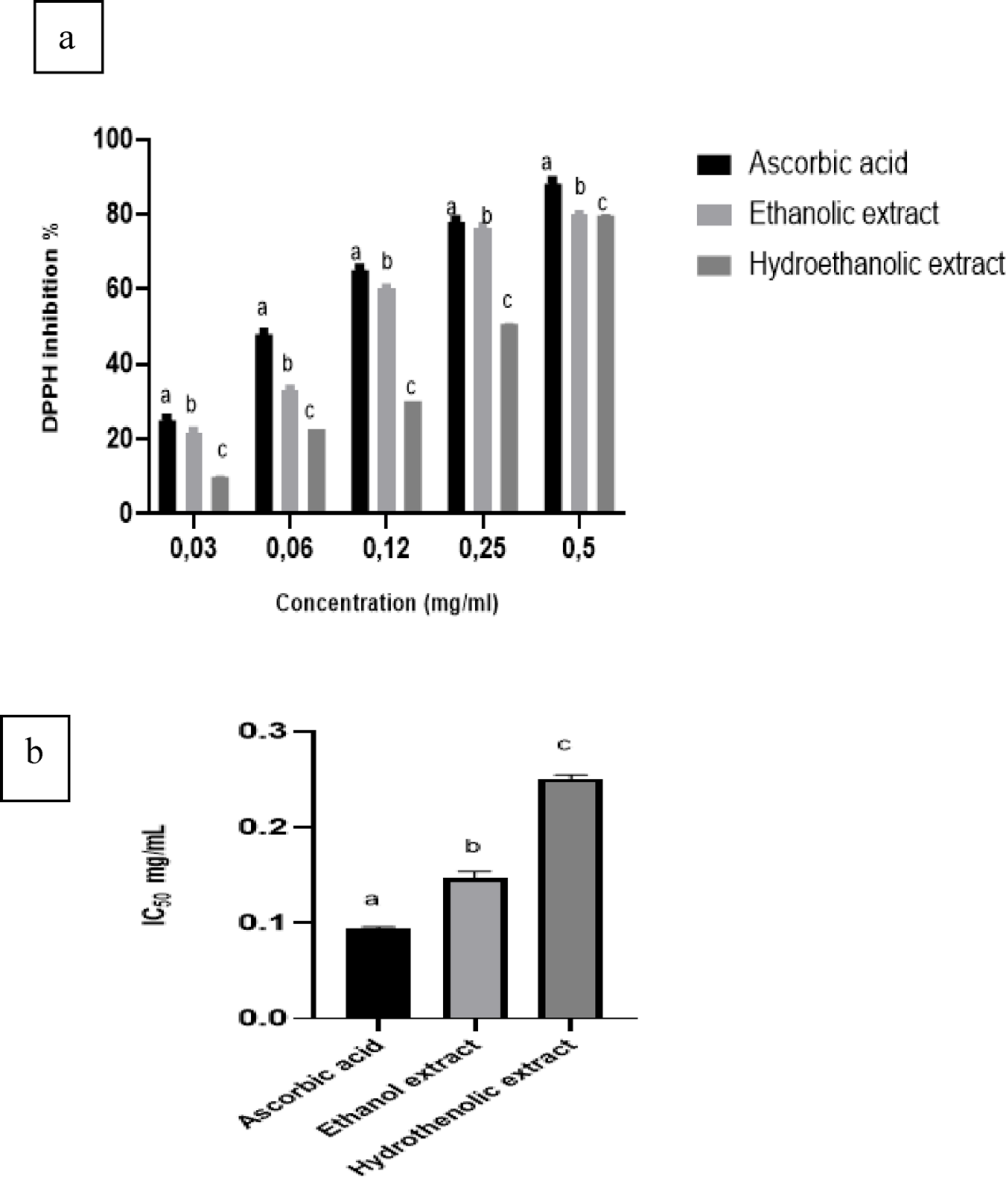
Antioxidant activity of sea buckthorn extracts as assessed by DPPH assay. (**a**) Scavenging percentage (% inhibition), (**b**) IC_50_ values (mg/mL).

The antioxidant capacity of sea buckthorn leaf extracts, evaluated by DPPH and FRAP assays, demonstrated a dose-dependent response for both solvents. Notably, the ethanolic extract exhibited activity of extracts was evaluated using the percentage scavenging activities and IC_50_ values (mg/mL). Results showed that both extracts exhibited antioxidant activity. In addition, ethanolic extract was more potent in the DPPH assay as its IC_50_ was lower (0.147 mg/mL) than that of the hydroethanolic extract (0.250 mg/mL), as shown in Fig. 3.

Results represent the mean of three independent replicates (n = 3). Values with different letters (a, b, c) within the same concentration or group have significant difference at $p\le 0.05$ according to one-way ANOVA and Tukey’s post-hoc test.

Furthermore, the antioxidant capacity was dose-dependent, with a scavenging percentage of 80% at the highest tested concentration of 0.5 mg/mL for both the hydroethanolic and ethanolic extracts. While hydroethanolic extracts showed higher total phenolic content, the ethanolic extract was more effective in the DPPH assay, which could be explained by the specific phenolic compounds extracted more effectively by the pure ethanol solvent, despite the overall lower yield. However, the reducing power of the two extracts was found to be in a dose-dependent manner. The results were in agreement with the study by^38^, in which strong antioxidant activity was observed, with similar antioxidant profiles (scavenging percentage of 70% at 0.05 mg/mL). Another study by^39^ reported strong free radical scavenging activity of the hydroethanolic extract of a milled fruits and leaves of sea buckthorn with an IC_50_ value of 0.07 mg/mL. An additional measure of antioxidant activity is the reducing power. Ferric reducing activity values of EC_50_ were respectively 0.22 and 0.34 mg/mL for the ethanolic and hydroethanolic extracts. Altogether, these results are in line with those obtained by^40^, who reported that sea buckthorn leaves presented the highest yield of extraction, contained much more TPC, while it showed an intermediate FRAP value.

### Antimicrobial activity

As shown in Table 2, the results of the inhibition tests, carried out by the agar well diffusion method, revealed significant antibacterial activity of the Algerian sea buckthorn leaf extracts against both *E. coli and S. aureus*. The validity of the assay was confirmed by the positive control, gentamicin, which produced inhibition zones of 17.29 ± 0.004 mm for *S. aureus* and 20.62 ± 2.33 for *E. coli.* The strongest antibacterial activity was exhibited by the hydroethanolic extract against the Gram-positive *S. aureus* strain, with an inhibition zone of 14.37 mm, compared to 10.47 mm for the ethanolic extract. However, antimicrobial activity against the Gram-negative *E. coli* was similar for both solvents, with inhibition zones of 10.20 mm and 10.38 mm for the hydroethanolic and ethanolic extract, respectively. This result is consistent with previous findings, such as those reported by^31^ who demonstrated the effectiveness of hydroethanolic sea buckthorn leaf extracts against these two pathogens*.* Similarly, the growth inhibitory activity of the ethanolic leaf extracts was confirmed by^33^ against the two studied strains. However, some previous research reported weak activity against *S. aureus* and no inhibitory effect against *E. coli* for sea buckthorn leaf extracts^41^. In contrast, our results align with more recent evaluations where 20% hydroethanolic extracts of sea buckthorn young leaves showed significant activity against both Gram-positive and Entrobacteriaceae strains^42^. To evaluate if this antimicrobial potency was quantitatively linked to the phytochemical profile, a Pearson correlation analysis was performed. For the hydroethanolic extract, a strong postive correlation was observed between the phenolic classes and the inhibition zones of *S. aureus*, with a correlation coefficient of *r* = 0.9934. Although this correlation was statistically not significant (*p* = 0.0732), it suggests that the phenolic fraction is the main driver of the antimicrobial response. In contrast, the correlation for *E. coli* was notably lower (*r* = 0.5000, *p* = 0.6667). This lack of significance further highlights that the activity is likely governed by the specific chemical structures and synergistic effects of the identified molecules, rather than a simple dose–response relationship of total contents. This is consistent with the findings of^42^, who reported that antimicrobial activity in sea buckthorn depends on the specific chemical structure of each polyphenol rather than its total concentration.

**Table 2:** Antimicrobial activity (Zone of inhibition) of sea buckthorn extracts and standard antibiotics.

Extract/antibiotics	*S. aureus* (mm)	*E. coli* (mm)
Ethanolic extract	10.47 ± 0.85^a^	10.38 ± 1.12^a^
Hydroethanolic extract	14.37 ± 0.94^b^	10.20 ± 0.76^a^
Gentamicin (positive control)	17.29 ± 0.004^d^	20.62 ± 2.33^c^
Ethanol (negative control)	0.00 ± 0.00^e^	0.00 ± 0.00^e^

Values are expressed as mean ± SD (n = 3).

Values with different superscripts within the same column are significantly different (*p* < 0.05) according to Tukey’s post-hoc test.

The in vitro antimicrobial potency observed in this study, especially against *S. aureus* is interesting, and can be explained by the complex phytochemical profile of the Algerian wild species. Specifically, the antibacterial activity of the extract is not solely dependent on a single class but on the synergy between flavonoids and tannins. The latter are known to directly damage bacterial membranes or interact with cell wall proteins^43–45^. It is biologically relevant that S*. aureus* is more susceptible than *E. coli*, and this is probably due to the structural differences between Gram-positive and Gram-negative cell walls^46^. The lack of an outer lipopolysaccharide membrane in *S*. *aureus* may facilitate the diffusion of the identified phenolic aglycones to their molecular targets, including PBP3. On the other hand, the outer membrane of *E. coli* acts as a selective permeability barrier, which may restrict the intracellular concentration of the bioactive compounds and cause the observed variations in inhibitory zones. The significant inhibition zones observed against *S*. *aureus* is correlated with the strong binding affinities by our molecular modeling results. Specifically, the high binding affinity of myricetin towards the PBP3 transpeptidase domain (7ONN) suggests a significant disruption of bacterial cell wall synthesis, thereby providing a mechanistic explanation of the observed zones of inhibition. While we acknowledge that agar well diffusion is a semi-quantitative method, in which some factors like diffusion rate of phenolics in the agar can influence the results, the clear correlation with our molecular docking scores provide valuable susceptibility profile. These findings serve as primary screening tools for future quantitative assays. Consequently, further studies focusing on the determination of quantitative antimicrobial indices are required to establish the efficacy and the pharmacological potency of the extract.

### Individual phenolic compounds analysis

**Fig. 4:**
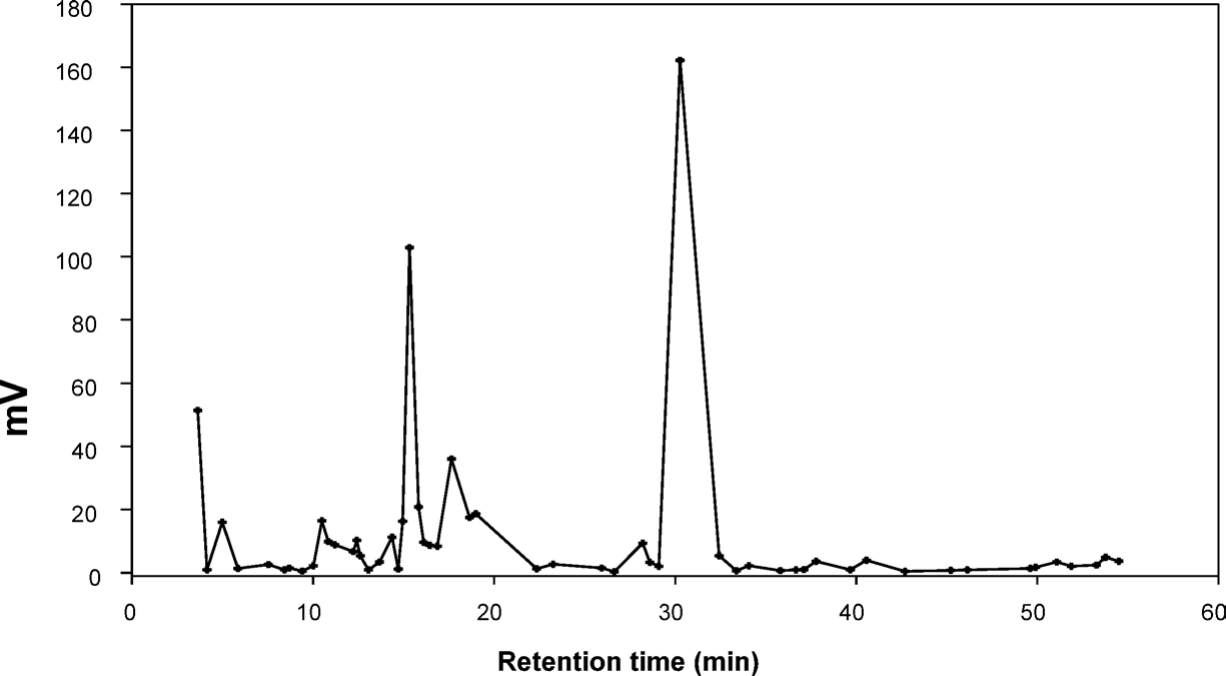
HPLC chromatogram of the sea buckthorn hydroethanolic leaf extract.

From the above results, it is clear that the hydroethanolic extract was the highest in terms of natural compounds, antioxidant and antimicrobial activities, in comparison with the ethanolic extract. Thus, HPLC analysis was performed to identify and quantify the phenolic compounds and ascorbic acid in the hydroethanolic leaf extract, and the results are presented in Table 3. The most abundant phenolic compounds among those identified (10 compounds) in the sea buckthorn leaf hydroethanolic extracts were catechin (72.35) and myricetin (6.34), while vanillin, kaempferol and gallic acid were minor fractions (Fig. 4, Table 3).

**Table 3:** Chemical properties and content of identified compounds from hydroethanolic extract leaves.

	Name	Formula	Molecula r weight(g ·mol^-1^)	Retention time (min)	Area (%)	Height (mV)	Content (µg/g DW)
01	Ascorbic acid	^C^_6_^H^_8_^O^_6_	176.12	3.64	0.84	51.45	1.04 ± 0.24
02	Gallic acid	^C^_7_^H^_6_^O^_5_	170.12	7.54	0.51	2.59	0.94 ± 0.14
03	Vanillin	^C^_8_^H^_8_^O^_3_	152.15	12.23	0.49	6.76	0.60 ± 0.23
04	Caffeic acid	^C^_9_^H^_8_^O^_4_	180.16	14.95	1.19	16.29	2.22 ± 0.32
05	Myricetin	^C^_15_^H^_10_^O^_8_	318.23	17.65	3.38	36.02	6.34 ± 0.96
06	Coumarin	^C^_9_^H^_6_^O^_2_	146.14	18.64	5.36	17.54	5.76 ± 3.43
07	Epicatechin	^C^_15_^H^_14_^O^_6_	290.27	18.99	6.17	18.71	5.88 ± 4.80
08	Kaempferol	^C^_15_^H^_10_^O^_6_	286.24	23.25	0.76	2.69	1.34 ± 0.14
09	Catechin	^C^_15_^H^_14_^O^_6_	290.27	30.27	41.02	162.17	72.35 ± 6.34
10	Tannic acid	^C^_76_^H^_52_^O^_46_	1701.2	10.50	1.62	16.45	3.01 ± 0.43

Indeed, catechin represents more than 50% of the phenolics identified in sea buckthorn leaves extract. Lower amounts of caffeic acid, ascorbic acid and kaempferol were identified. These compounds have been reported in previous studies with some similar results, such as^47^ who reported that the most abundant flavonoid was epicatechin. Ellagitannin was previously reported by^48–50^. Flavonoids from sea buckthorn leaves have been previously reported^51^, among them, two compounds were detected in the sea buckthorn extract samples as quercetin and kaempferol.^52^ reported that sea buckthorn leaves extracts analyzed by RP-HPLC contained in addition to these compounds, gallic acid, isorhamnetin, and myricetin. Gallic acid and myricetin were also detected in the hydroethanolic extract of the sea buckthorn leaves of the current research study. However,^53^ reported that myricetin was detected only in whole berries but not leaves.^54^ reported that the main constituents are gallic acid, rutin, quercetin-3-galactoside, quercetin-3-glucoside, myricetin, quercetin, kaempferol and isorhamnetin.^31^ identified also two quercetin derivatives, kaempferol and isorhamnetin in the aqueous and hydroethanolic leaf extract. Furthermore, the HPLC profile of sea buckthorn leaves from Romanian varieties identified 14 phenolic compounds, including 9 flavonoids, 4 cinnamic acid derivatives and one gallic acid, in which quercitrin was the most abundant phenolic compounds in all leaf samples^7^. The quantitative analysis revealed high concentrations of catechin and myricetin. The biological relevance of these concentrations is concerned by the high internal potential of these aglycones. Myricetin, although at low concentrations, has been well reported to exhibit significant antimicrobial effects by inhibiting bacterial ATP synthase and disrupting membrane integrity. Furthermore, while some compounds like gallic acid and Kaempferol, were relatively low, their detection is relevant as they often act synergistically with major components, enhancing the overall antioxidant and antibacterial effects of the extracts. Furthermore, the presence of specific aglycones like myricetin (6.34) in this wild population is significant, as these molecules possess high intrinsic reactivity and bioavailability compared to the glycosylated forms often reported in other geographic regions.

### Binding affinity analysis

**Fig. 5:**
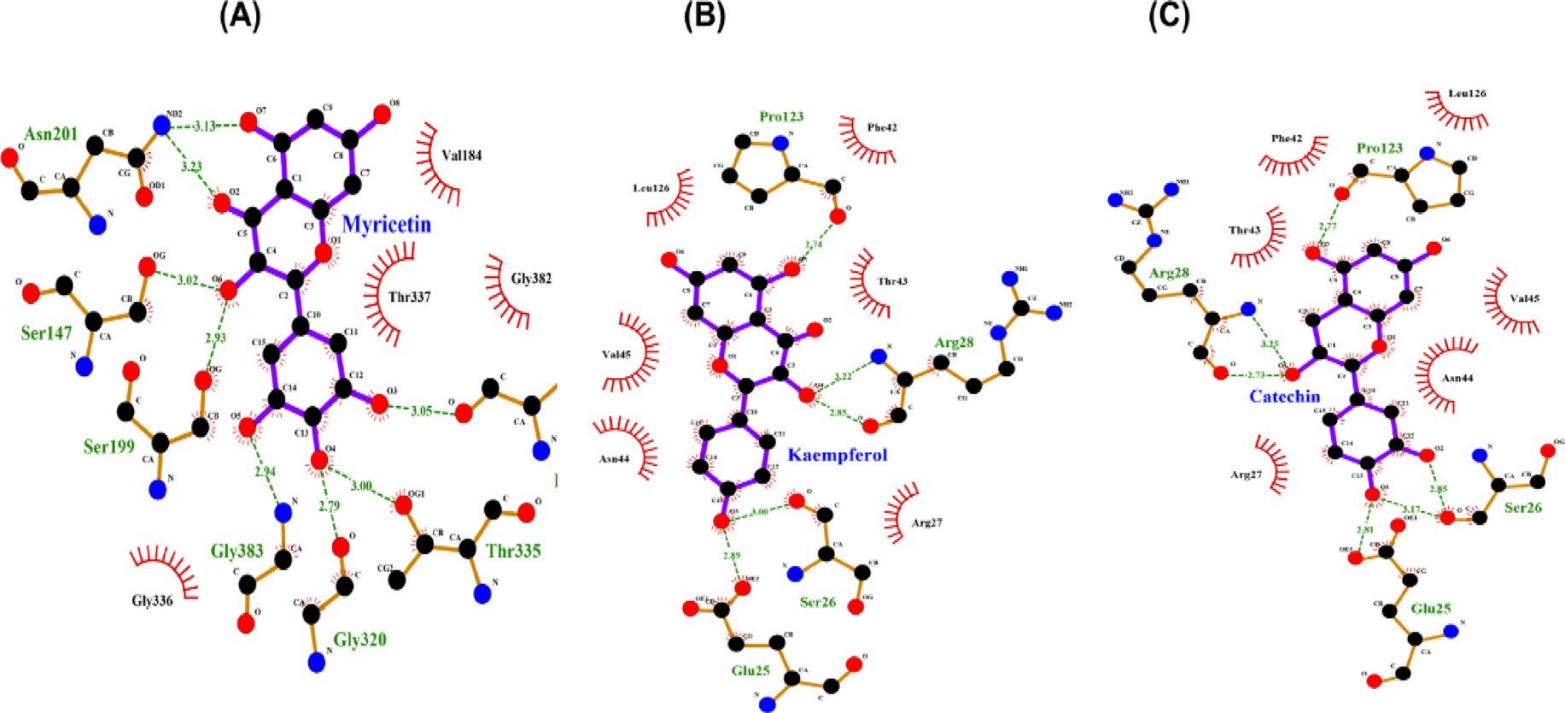
Molecular docking interactions of the top-ranked phytochemicals within the 7ONN binding pocket. 2D interaction diagrams for: (**A**) Catechin, (**B**) Kaempferol, and (**C**) Myricetin. Green doshed lines represent hydrogen bonds, while red semi-circles indicate hydrophobic interactions with surrounding amino acid residues.

To bridge the gap between phytochemical profiling and the observed in vitro growth inhibition, we performed a targeted in silico screening against the PBP3 transpeptidase domain (7ONN), an essential enzyme for bacterial septation. We hypothesized that the antimicrobial activity is mediated by the targeted inhibition of this cell wall synthesis enzyme. While HPLC analysis identified 10 bioactive compounds, we prioritized those with the highest content (Catechin) and those exhibiting the highest predicted chemical reactivity in our DFT study (Myricetin and Kaempferol). By focusing on these specific bioactive compounds, we aim to understand how sea buckthorn leaf extracts act at the molecular level against pathogens like *S. aureus.* The results in the Table 4 demonstrate notable differences in the binding free energy values of the selected ligands (Myricetin, Kaempferol, and Catechin) with the target proteins 7ONN (Fig. 5). The ligands exhibiting the strongest binding affinities include *Myricetin* (− 8.1 kcal/mol), *Kaempferol* (− 7.8 kcal/mol), *Catechin* (− 7.7 kcal/mol), and *Epicatechin* (− 7.2 kcal/mol). These scores suggest robust interactions within the protein’s active site. Moderate affinities are observed for *Caffeic* acid (− 6.2 kcal/mol), *Ascorbic acid*, and *Coumarin* (both − 5.9 kcal/mol), while *Vanillin* showed the weakest interaction (− 5.3 kcal/mol). Overall, the data from the Table 4 suggest that ligands such as *Myricetin*, *Kaempferol*, *Catechin*, and *Epicatechin* have the strongest binding affinities with the target protein, making it promising candidates for further investigations. Conversely, *Vanillin* shows consistently weak interactions and is less likely to be a key contributor to the observed biological activity.

**Table 4:** Binding free energy and key molecular interactions of selected ligands with 7ONN.

Target protein	Ligand	Binding free energy (kcal/mol)	Key interacting residues	H-bond distances (Å)
7ONN	Ascorbic acid	− 5.9	Asn201, Ser199, Thr337, Ser147, Lys150, Gly146, Val184, Tyr259, Phe257	2.70–3.19
	Gallic acid	− 5.7	Thr335, Ser199, Tyr381, Ser147, Lys334, Thr337, Gly336, Gly383	2.80–3.29
	Vanillin	− 5.3	Ser199, Ser147, Tyr381, Lys334, Thr337, Thr335, Gly336	2.83–3.12
	Caffeic acid	− 6.2	Tyr380, Thr335, Ser199, Ser147, Lys334, Tyr381, Gly336, Gly383, Val184	2.81–3.28
	Coumarin	− 5.9	Tyr381, Lys334, Ser199, Gly320, Thr335, Gly336, Thr337	2.98
	Epicatechin	− 7.2	Gly320, Asn201, Thr335, Val184, Tyr381, Thr337, Gly383, Ser147, Ser199	2.80–3.12
	Kaempferol	− 7.8	Arg28, Glu25, Ser26, Asn44, Pro123, Thr43, Phe42, Leu126, Arg27, Val45	2.74–3.22
	Myricetin	− 8.1	Ser199, Asn201, Thr335, Gly320, Ser147, Thr337, Gly383, Gly336, Val184, Gly382	2.79–3.23
	Catechin	− 7.7	Arg28, Ser26, Val45, Asn44, Glu25, Pro123, Thr43, Leu126, Phe42, Arg27	2.73–3.25

### DFT analysis and its correlation with molecular docking results

**Fig. 6:**
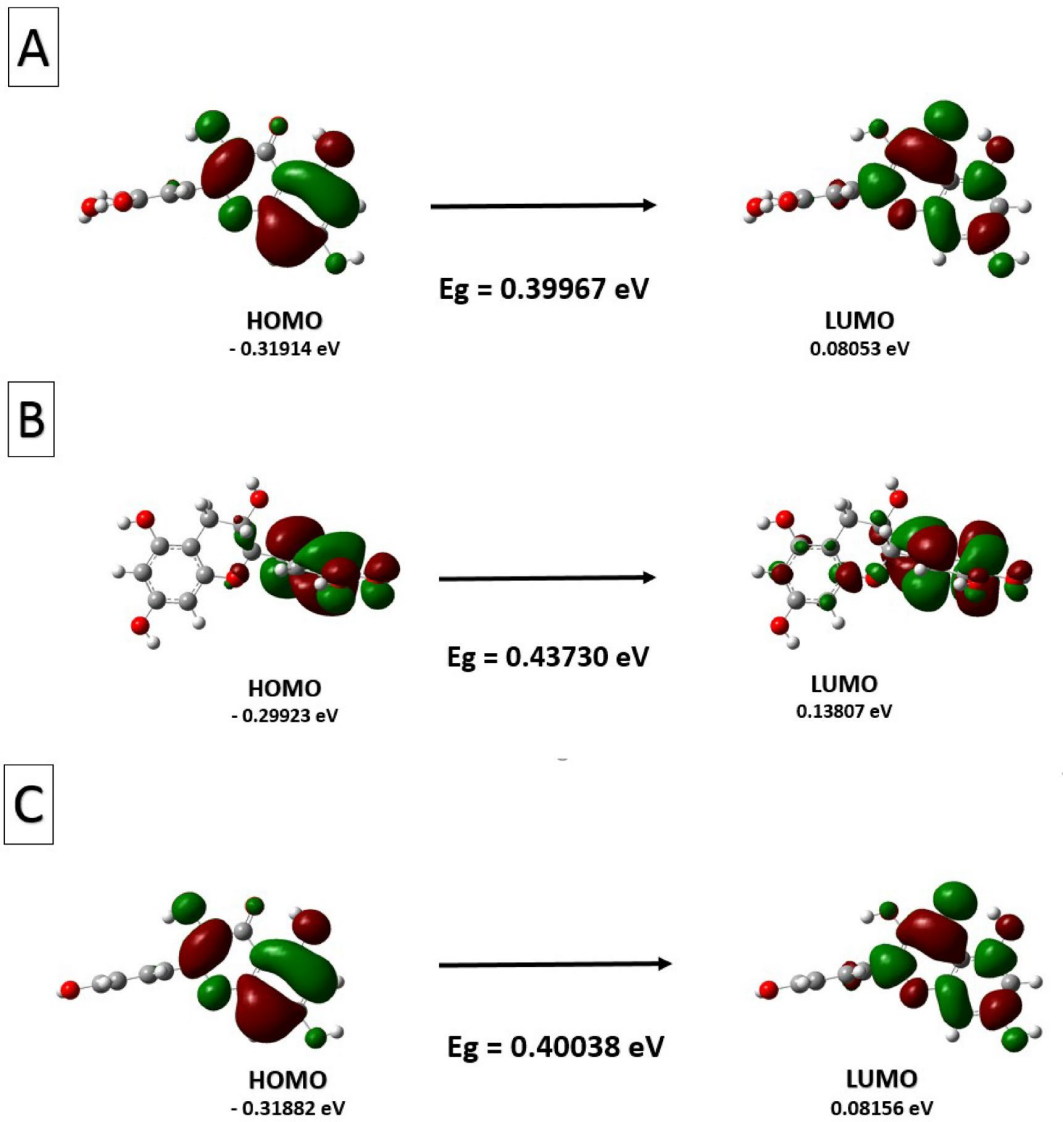
HOMO and LUMO orbital distributions of Myricetin (**A**), Catechin (**B**), and Kaempferol (**C**) with energy gaps (Eg).

**Fig. 7:**
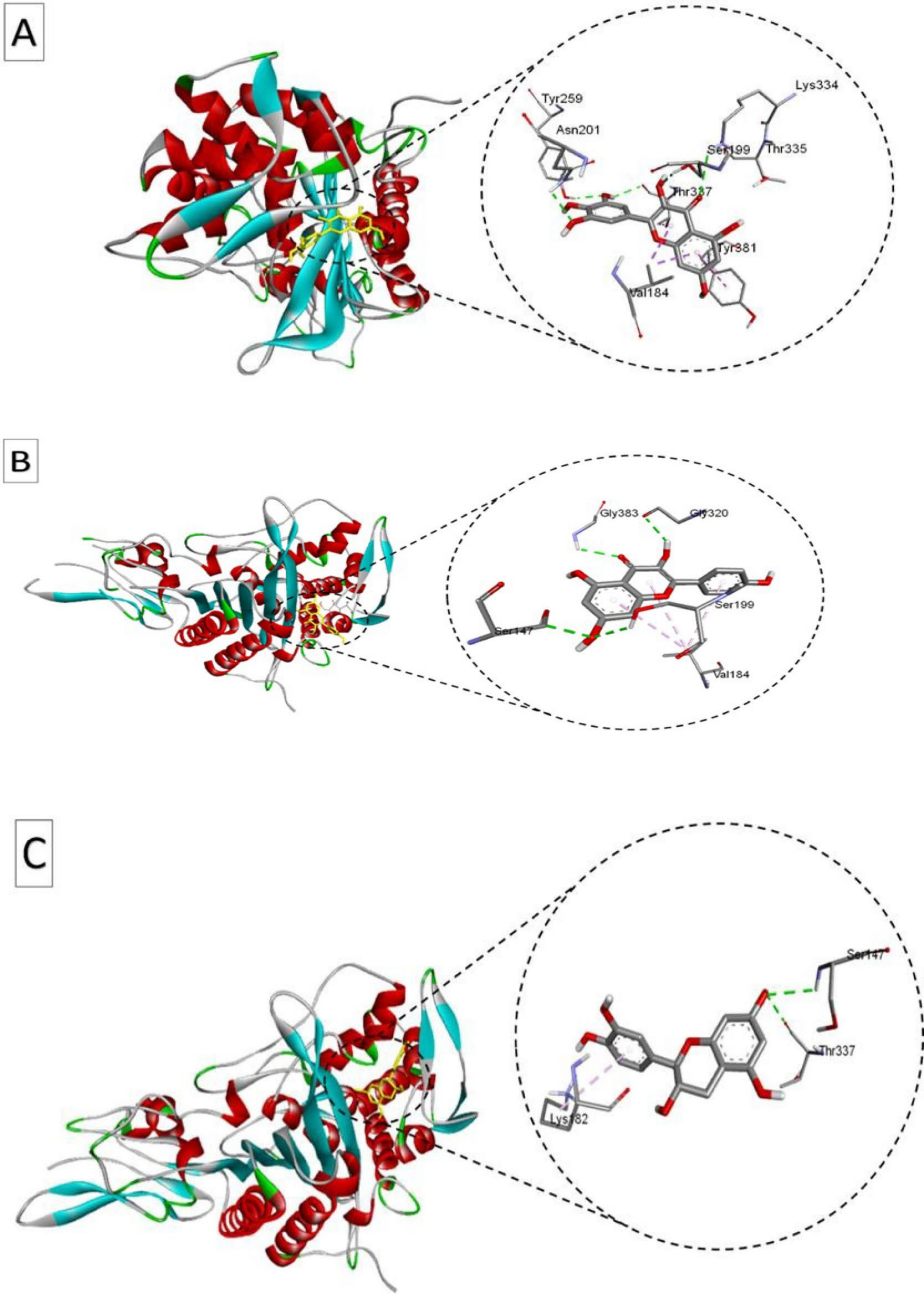
Binding interactions of selected ligands with 7ONN protein binding pocket. Detailed visualization of the molecular interactions for (**A**) 7ONN-Myricetin, (**B**) 7ONN-Kaempferol, and (**C**) 7ONN-Catechin highlighting key amino acid residue interactions, illustrating the spatial orientation and stabilization of these phytochemicals within the active site.

The results of the HOMO–LUMO energy gap calculations (Table 5 and Fig. 6) provide important perspectives into the chemical reactivity and stability of the selected ligands, directly correlating with their molecular docking performance. Ligands with smaller HOMO–LUMO gaps, such as *Caffeic acid* (0.37957 eV), *Vanillin* (0.39779 eV), and *Myricetin* (0.39967 eV), generally exhibit higher chemical reactivity^55^. Among these, *Myricetin* showed the strongest binding affinity with 7ONN protein (− 8.1 kcal/mol), suggesting a dense network of hydrogen bonds with residues Ser147, Asn201, and Thr335 (Figs. 5 and 7) direct link between its smaller energy gap and its high interaction potential^56^. This correlation between frontier orbitals and predicted biological activity is a cornerstone of Density Functional theory in chemical biology^12,14^. To provide a more comprehension characterization of molecular reactivity, global reactivity indices were further determined (Table 5). Myricetin and caffeic acid exhibited the lowest hardness (ɳ) values (0.199 and 0.189 eV respectively), indicating their high chemical reactivity and a propensity for charge transfer. Furthermore, the electrophilicity index $(\omega )$ values suggest that molecules such as coumarin and myricetin have a strong tendency to interact with electron-rich regions of the target protein. These results are highly consistent with the potent binding affinities observed in the docking study for the 7ONN target. Conversely, ligands with larger energy gaps, such as *Catechin* (0.43730 eV) and *Epicatechin* (0.43494 eV), demonstrated strong binding affinities despite their lower reactivity, with docking scores of − 7.7 and − 7.2 kcal/mol for 7ONN**,** respectively. This suggests that structural complementarity and specific hydrogen bonding networks, specially inetractions with Arg28, Ser26, and Glu25, significantly influence their interactions stability^26^. Ligands with moderate gaps, such as *Kaempferol* (0.40038 eV) and tannic acid (0.40365 eV), performed well in docking studies (− 7.8 kcal/mol for 7ONN), balancing reactivity and stability. In contrast, *Vanillin*, despite its smaller energy gap (0.39779 eV), showed weaker binding affinities (− 5.3 kcal/mol for 7ONN), emphasizing that chemical reactivity alone is not sufficient for strong protein–ligand interactions^57^. Overall, the correlation between docking results and HOMO–LUMO energy gaps highlights the importance of both chemical reactivity and structural features in determining ligand binding efficiency, with *myricetin*, *kaempferol* and *catechin* emerging as the strong candidates for further investigation.

**Table 5:** Global reactivity descriptors of the identified ligands from sea buckthorn.

Ligands	HOMO (eV)	LUMO (eV)	Gap (eV)	Global hardness (ɳ)	Chemical potential (µ)	Electrophilicity Index ($\omega$)
Ascorbic acid	− 0.34601	0.10550	0.45151	0.22575	− 0.12025	0.03203
Gallic acid	− 0.32578	0.08581	0.41159	0.20579	− 0.11998	0.03497
Vanillin	− 0.31385	0.08394	0.39779	0.19889	− 0.11495	0.03322
Caffeic acid	− 0.30470	0.07487	0.37957	0.18978	− 0.11491	0.03478
Myricetin	− 0.31914	0.08053	0.39967	0.19983	− 0.11930	0.03561
Coumarin	− 0.33077	0.06357	0.39434	0.19717	− 0.13360	0.04526
Epicatechin	− 0.30743	0.12751	0.43494	0.21747	− 0.08996	0.01860
Kaempferol	− 0.31882	0.08156	0.40038	0.20019	− 0.11863	0.03515
Catechin	− 0.29923	0.13807	0.43730	0.21865	− 0.08058	0.01484
Tannic acid	− 0.32115	0.08250	0.40365	0.20182	− 0.1192	0.03527

### Multitarget antibacterial potential

To broaden the understanding of the antibacterial mechanism of the lead compounds identified from Algerian wild sea buckthorn, two additional vital target proteins were selected for a comparative docking study. The selection of FtsZ (PDB ID: 1W5A) and DNA Gyrase B (PDB ID: 1KZN) was highly relevant to complement the study of PBP3 (7ONN), as these targets represent distinct vital pathways. While PBP3 is essential for cell wall synthesis, FtsZ is a key protein for bacterial cell division and DNA Gyrase B is crucial for DNA replication and structural maintenance. By evaluating these three distinct pathways, we aimed to determine whether the identified flavonoids, specifically Myricetin and Kaempferol, exert a multi-target inhibitory effect by simultaneously disrupting cell wall integrity, division, and genetic replication, thereby reducing the development of bacterial resistance. The comparative binding affinities and molecular interactions of these lead compounds across the three vital targets are summarized in Table 6. Myricetin and kaempferol both showed strong affinities for FtsZ, with binding energies of − 7.74 and − 7.67 kcal/mol, respectively. These values are similar to those obtained for PBP3, suggesting a dual inhibition of cell division and cell wall production. While the interactions with DNA Gyrase B were comparatively more moderate (ranging from − 5.26 to − 5.37 kcal/mol), the overall profile supports a synergistic multi-pathway mechanism.

**Table 6:** Comparative binding affinities of the most potent sea buckthorn flavonoids across multiple bacterial targets.

Target protein	Ligand	Binding Free Energy (kcal/mol)	H-bond residues	H-bond distances (Å)
FtsZ (1W5A)	Myricetin	− 7.74	Asp255, Asp259, Val319, Gln320	1.91–3.12
	Kaempferol	− 7.67	Asp217	2.23
DNA Gyrase B (1KZN)	Myricetin	− 5.26	Glu31, Asn32, Asp59	1.84–3.11
	Kaempferol	− 5.37	Ala14, Asp35, Asp59	1.84–2.37
PBP3 (7ONN)	Myricetin	− 8.10	Arg28, Glu25, Ser26, Asn44, Pro123, Thr43, Phe42, Leu126, Arg27, Val45	2.79–3.23
	Kaempferol	− 7.80	Ser199, Asn201, Thr335, Gly320, Ser147, Thr337, Gly383, Gly336, Val184, Gly382	2.74–3.22

Among the three proteins evaluated, the PBP3 (7ONN) transpeptidase domain exhibited the most consistent and highest binding scores for both ligands. To validate these static docking predictions and ensure the long-term stability of the interactions, 100 ns Molecular Dynamics (MD) simulations were subsequently performed on the 7ONN-ligand complexes.

Molecular dynamics simulations results.

**Fig. 8:**
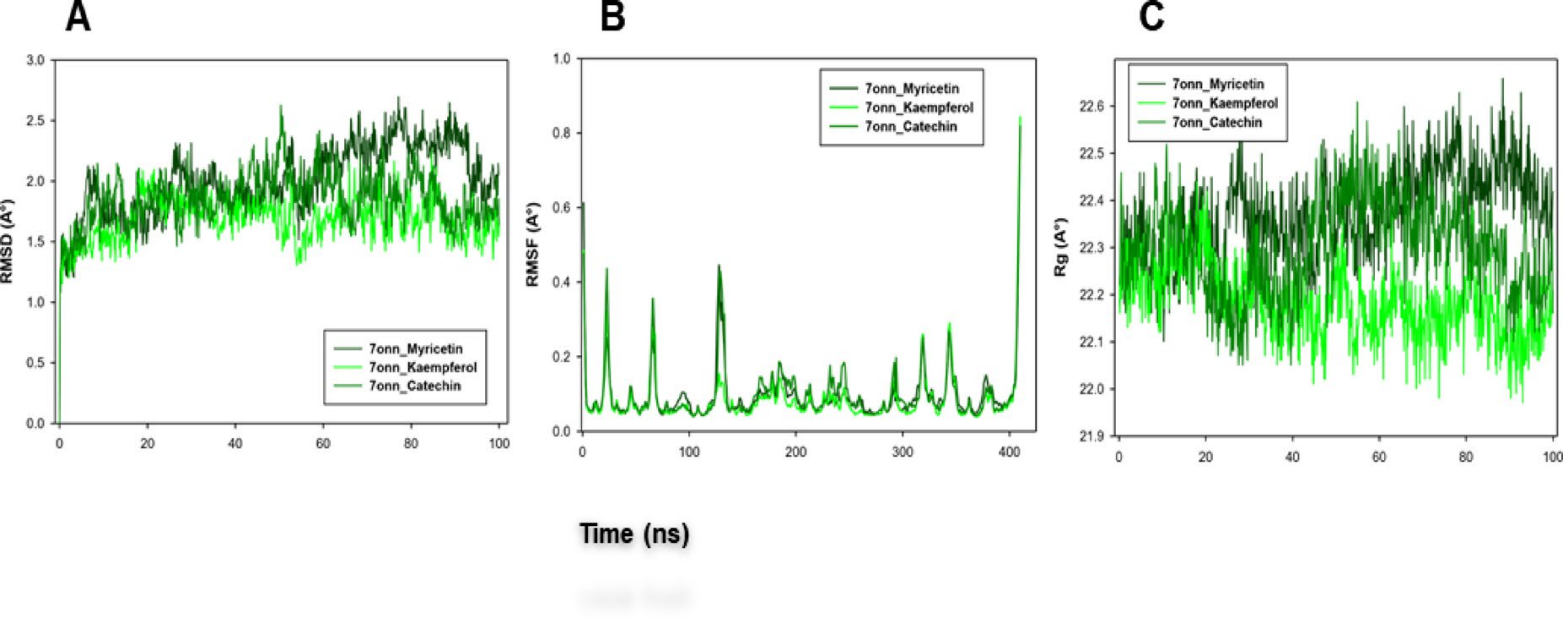
Molecular dynamics (MD) simulation analysis of the 7ONN protein–ligand complexes. The figure displays the stability and fluctuation parameters over a 100 ns trajectory: (**A**) Root Mean Square Deviation (RMSD), (**B**) Root Mean Square Fluctuation (RMSF), and (**C**) Radius of Gyration (*R*_g_).

The stability and flexibility of the 7ONN complexes involving Myricetin, Kaempferol, and Catechin were evaluated through 100 ns molecular dynamics simulations. Root-mean-square deviation (RMSD) analysis revealed that the 7ONN- Kaempferol complex exhibited the lowest deviations, stabilizing at 1.5–2.0 Å, indicative of strong and stable binding. In contrast, the 7ONN-Myricetin complex showed higher RMSD values (2.0–2.5 Å) and greater fluctuations, suggesting slightly reduced stability (Fig. 8A). Desptite myricetin has higher docking score, the 100 ns reveals that Kaempferol establishes a more robust and steady hydrogen bonding network. The stable (RMSD, ~ 1.5–2.0 Å) and low RMSF < 0.8 Å) of the 7ONN-kaempferol complex reconcile the initial affinity predictions with long-term structural stability under dynamic conditions.

Similarly, root-mean-square fluctuation (RMSF) analysis demonstrated that all complexes maintained similar residue flexibility patterns, with minimal fluctuations (< 0.8 Å) for most binding site residues, except for loop regions and the C-terminal end, where peaks near residue index ~ 400 were observed (Fig. 8B). Lastly, radius of gyration (Rg) analysis confirmed that all complexes retained consistent compactness (22.2–22.8 Å) throughout the 100 ns simulation, with the 7ONN_Kaempferol complex being slightly more compact (Fig. 8C). Collectively, these findings suggest that Kaempferol forms the most stable and compact complex, followed by Catechin, while Myricetin demonstrates reduced stability under dynamic conditions. This mechanistic approach demonstrates how specific aglycones from sea buckthorn leaves effectively disrupt the bacterial cell wall assembly, providing a molecular-level explanation for the growth inhibition observed in the antimicrobial assays. RMSD analysis revealed that all complexes reached equilibrium within the first 20 ns, maintaining overall structural integrity with values below 3.0 Å. Although this integrated in silico approach, combining molecular docking, DFT, and MD simulations, successfully identified high-affinity interactions, the pharmacological potential of the major identified compounds is further supported by recent in silico studies. Predictions of ADMET (Absorption, Distribution, Metabolism, Excretion, and Toxicity) and PASS (Prediction of Activity Spectra for Substances) analyses have demonstrated that these bioactive molecules exhibit high-drug-likeness and low toxicity profiles, reinforcing their therapeutic potential. These predictive tools provide crucial insights to validate the safety and effectiveness of the compounds, offering a deeper understanding of the observed biological properties and providing support for both in silico and in vitro findings^15,58^.

## Conclusions

This study provides a comprehensive phytochemical and mechanistic potential of wild sea buckthorn (*Hippophae rhamnoides* L.) leaf extracts from the Algerian coast. While the ethanolic extract exhibited higher antioxidant capacity (DPPH assay), the hydroethanolic extract yielded a higher total phenolic content and more potent antimicrobial activity against *S. aureus*. This suggests that antimicrobial potency is closely linked to total phenolic density, whereas antioxidant power is attributed to specific bioactive molecules more effectively solubilized in pure ethanol. Catechin, myricetin, and kaempferol were identified as the key bioactive compounds. Furthermore, these phenolics demonstrated antibacterial action, supported by high binding affinities across multiple targets and validated by the structural stability of the PBP3 -kaempferol complex (7ONN) through high-precision 100 ns MD simulations. The integration of advanced computational tools successfully bridged the gap between phytochemical profile and biological mechanisms. The multi-target approach revealed that the identified flavonoids exhibit a synergistic effect, which is particularly relevant in the context of overcoming antibiotic resistance. While the bacterial transpeptidase PBP3 was confirmed as a primary stable target for cell wall disruption, the strong binding affinities observed with FtsZ (1W5A) and DNA Gyrase B (1KZN) suggest the simultaneous inhibition of bacterial cell wall synthesis, septation, and DNA replication.

Despire these promising results, it is important to note the limitations of the study. While the agar diffusion assay provides a reliable comparative screening, it remains a semi-quantitative method. Future research should focus on a broader panel of multi-drug resistant clinical isolates and conduct in vivo toxicity assessments to ensure pharmacological safety.

Overall, this research study highlights Algerian wild sea buckthorn leaves as a valuable and sustainable source of natural bioactive compounds with high potential for pharmaceutical and functional food applications.

## Supplementary Information

Below is the link to the electronic supplementary material.

Supplementary Material 1

Supplementary Material 2

Supplementary Material 3

## Data Availability

All data generated or analysed during this study are included in this published article.
