# Supplementary material for: Phytochemical profiling and multi-target antibacterial in silico potential of Algerian wild sea buckthorn leaves
Source: Sci Rep. 2026 Jun 21;16:19359. doi: 10.1038/s41598-026-57022-2 (PMC13287622; doi:10.1038/s41598-026-57022-2)
Supplement: Supplementary file 1 — Supplementary Material 1 [file 41598_2026_57022_MOESM1_ESM.docx]

|  | **Name** | **Formula** | **Molecular Weight(g·mol^-1^)** | **Retention time (min)** | **Area (%)** | **Height**  **(mV)** | **Content**  **( µg/g DW)** | **R^2^** | **LOD**  **(µg/mL)** | **LOQ**  **(µg/mL)** |
| --- | --- | --- | --- | --- | --- | --- | --- | --- | --- | --- |
| 01 | Ascorbic acid | C6H8O6 | 176.12 | 3.64 | 0.84 | 51.45 | 1.04±0.24 | 0.995 | 0.20 | 0.65 |
| 02 | Gallic acid | C7H6O5 | 170.12 | 7.54 | 0.51 | 2.59 | 0.94±0.14 | 0.998 | 0.15 | 0.50 |
| 03 | Vanillin | C8H8O3 | 152.15 | 12.23 | 0.49 | 6.76 | 0.60±0.23 | 0.996 | 0.35 | 1.50 |
| 04 | Caffeic acid | C9H8O4 | 180.16 | 14.95 | 1.19 | 16.29 | 2.22±0.32 | 0.997 | 0.25 | 0.80 |
| 05 | Myricetin | C15H10O8 | 318.23 | 17.65 | 3.38 | 36.02 | 6.34±0.96 | 0.999 | 0.10 | 0.35 |
| 06 | Coumarin | C9H6O2 | 146.14 | 18.64 | 5.36 | 17.54 | 5.76±3.43 | 0.995 | 0.40 | 1.30 |
| 07 | Epicatechin | C15H14O6 | 290.27 | 18.99 | 6.17 | 18.71 | 5.88±4.80 | 0.997 | 0.28 | 0.95 |
| 08 | Kaempferol | C15H10O6 | 286.24 | 23.25 | 0.76 | 2.69 | 1.34±0.14 | 0.996 | 0.32 | 1.05 |
| 09 | Catechin | C15H14O6 | 290.27 | 30.27 | 41.02 | 162.17 | 72.35±6.34 | 0.999 | 0.05 | 0.18 |
| 10 | Tannic acid | C76H52O46 | 1701.2 | 10.50 | 1.62 | 16.45 | 3.01±0.43 | 0.995 | 0.48 | 1.60 |

Table 2 : Chemical properties and content of identified compounds from hydroethanolic extract leaves
