# Supplementary material for: Phytochemical profiling and multi-target antibacterial in silico potential of Algerian wild sea buckthorn leaves
Source: Sci Rep. 2026 Jun 21;16:19359. doi: 10.1038/s41598-026-57022-2 (PMC13287622; doi:10.1038/s41598-026-57022-2)
Supplement: Supplementary file 2 — Supplementary Material 2 [file 41598_2026_57022_MOESM2_ESM.docx]

**(A)**

**(B)**

**(C)**

**(D)**

**(E)**

**(F)**

Supplementary Figure S1. Chemical structures of additional phytochemical compounds identified in Algerian sea buckthorn leaves.

The figure displays the 2D structures of secondary matabolites used for the comparative molecular docking study : (Gallic acid), (B) Ascorbic acid, (C) Vanillin, (D) caffeic acid,, (E) Coumarin, and (F) Epicathechin. These strucrtues, complement the main bioactive markers.
